# Psychological trauma and post-traumatic growth in parents of children with sickle cell disease

**DOI:** 10.1016/j.heliyon.2024.e34283

**Published:** 2024-07-10

**Authors:** Ali Alsaad, Abdullah Alghanim, Mohammed Aldawood, Ali Al Zaid, Hussain Aldehneen, Rawan Aldrees, Ammar Alsalem, Sami Albattat, Abbas Al Mutair

**Affiliations:** a Department of Clinical Neurocience, King Faisal Universiry, Alahsa, Eastren Province of Saudi Arabia, Saudi Arabia; b Department of Psychiatry, College of Medicine, Imam Abdulrahman Bin Faisal University, Dammam, Saudi Arabia; c Department of Neurology, King Fahad Specialist Hospital, Dammam, Eastern Province of Saudi Arabia, Saudi Arabia; d Department of Urology, King Fahad Specialist Hospital, Dammam, Eastern Province of Saudi Arabia, Saudi Arabia; e College of Medicine, King Faisal Universiry, Alahsa, Eastren Province of Saudi Arabia, Saudi Arabia; f Department of Emergency medicine, King Fahad Hospital, Alahsa, Eastren Province of Saudi Arabia, Saudi Arabia; g Department of Pediatric Hematology, Maternity and Children Hospital, Alahsa, Eastren Province of Saudi Arabia, Saudi Arabia; h Research Center, Almoosa Specialist Hospital, Al-Hasa, Saudi Arabia; i School of Nursing, University of Wollongong, Wollongong, Australia; j Department of Medical-Surgical Nursing, College of Nursing Princess Nourah Bint Abdulrahman University, Riyadh, Saudi Arabia; k Department of Nursing, Prince Sultan Military College of Health Sciences, Dahran, Saudi Arabia; l Almoosa College of Health Sciences, Al-Ahsa, Saudi Arabia

**Keywords:** PTSD, PTG, Pediatric chronic Illnesses, Parenting, Sociodemographic factors, Sickle cell disease

## Abstract

Sickle cell disease (SCD) is a hereditary blood condition characterized by abnormal hemoglobin, leading to chronic hemolysis and vaso-occlusive complications. Caregivers of children with SCD often experience significant distress, akin to post-traumatic stress disorder (PTSD). This study aimed to measure the degree of trauma and post-traumatic growth among parents (caregivers) of children with SCD in the Kingdom of Saudi Arabia. A total of 294 primary caregivers were recruited for this study, through direct phone calls and online outreach using contact information obtained from their primary treating physician in Maternity and Children Hospitals and the Hereditary Blood Diseases Center in Al-Ahsa. Inclusion criteria required caregivers not to be receiving professional mental health care and to have a child with SCD below the age of 18. Results indicate that caregiver gender significantly affected IESR scores, with mothers reporting higher scores than fathers. Family income had a significant effect on IESR as well. In terms of education level, higher-educated caregivers were less likely to experience severe trauma. Significant differences emerged between online and phone interview participants, with online respondents reporting higher post-traumatic growth and higher trauma levels. This study represents a crucial step in understanding the challenges faced by caregivers of children with SCD in Al-Ahsa, Saudi Arabia. However, the study has limitations, including a substantial portion of the sample being from a single clinic and a cross-sectional design.

## 1 Introduction

Sickle cell disease (SCD) is a hereditary blood condition passed down from parents to children in an autosomal recessive pattern. It is characterized by the presence of an abnormal type of hemoglobin in affected individuals. Hemoglobin, a protein found in red blood cells, is responsible for transporting oxygen throughout the body. SCD affects this process of oxygen transportation to organs and tissues [1].

Individuals with SCD can present clinically with chronic hemolysis, increased susceptibility to infections, and vaso-occlusive complications, often requiring medical care [2]. Symptoms such as paleness and fatigue due to hemolytic anemia occur in all significant forms of SCD. The lifespan of red cells in HbSS patients is estimated to be around 17 days, compared to 120 days in healthy individuals [3,4]. Sickled cells can become sticky as they flow through small blood vessels, occluding these vessels and resulting in sharp pain. This pain can occur anywhere in the body, but the most common sites are the chest, arms, and legs [5,6].

Children with SCD get admitted to hospitals for various reasons. The most common cause of admissions worldwide is the acute painful crisis [7,8]. However, in developing countries, infections remain the top cause of admission [8].

SCD affects millions globally, with a high prevalence among descendants of sub-Saharan Africa, Spanish-speaking regions of the hemisphere (South America, the Caribbean, and Central America), Saudi Arabia, India, and Mediterranean countries such as Turkey, Greece, and Italy [9]. Studies have shown that SCD is a comparatively common genetic disorder in Saudi Arabia, with a range of 2 %–27 % in the carrier state and up to 1.4 % in the disease state [9]. Moreover, caregivers of children with chronic diseases generally experience high levels of distress that can lead to dysregulation of the hypothalamic-pituitary-adrenal axis and reduce their life span [10]. Given the significant impact of SCD on quality of life and the frequent hospitalizations due to various crises and complications, it is unsurprising that studies report that four out of ten caregivers experience emotional distress due to their child having SCD [11].

Furthermore, due to some lethal complications of SCD, it can be assumed that a caregiver of a child with the disease may experience elements of post-traumatic stress disorder (PTSD). PTSD can result from extremely stressful, distressing, or frightening events and is characterized by nightmares and flashbacks related to the event, accompanied by feelings of isolation, guilt, and irritability [12].

PTSD is clinically diagnosed using specific diagnostic criteria. These include exposure to the traumatic event (TE), involuntary memories of the TE, distressing dreams related to the TE, flashbacks of the TE, intense psychological and physiological reactions to cues symbolizing the TE, avoidance of stimuli related to the TE, and negative alterations in arousal, mood, and cognitions related to the TE [13].

Some studies have demonstrated the psychological impact of SCD on affected children and their parents. For instance, a study conducted in France, including SCD children and their parents, showed the presence of PTSD in SCD children and their parents similar to the findings found in cancer survival children and their families [14].

In addition, psychological challenges are widespread among the public, and studies indicate that levels of depression are particularly elevated among caregivers of children with cancer [15,16].

To the best of our knowledge, there are very few studies on PTSD among parents of children with SCD globally, with no study done in the region. This creates a knowledge gap in the literature regarding this issue. So, this study aims to measure the degree of trauma among caregivers (i.e., parents) of children affected with SCD, as well as post-traumatic growth. We plan to collect data through two methods, i.e., phone calls and online questionnaires, to identify any significant difference between them, especially after the COVID era when everything is being done with the least amount of friction and connection.

Finally, this study will evaluate the factors contributing to the degree of trauma (i.e., rate of admission, level of income, level of education) as PTSD in caregivers may affect the quality of care, causing more suffering in SCD patients and daily life disturbances. This study may help policymakers in the Kingdom of Saudi Arabia to identify the most vulnerable group and design supporting programs. Although this study sample is limited to the eastern region of Saudi Arabia, the results can be generalized globally due to the similarity in disease presentation and burden.

## 2 Methods

This study was conducted in accordance with the ethical standards outlined in the Declaration of Helsinki and was approved by the Institutional Review Board (IRB) under the ethical approval number 64-EP-2021.

### 2.1 Sample

This study involved 294 caregivers of children with sickle cell disease in the period between 2021 and 2022. The caregivers were conveniently invited to the study by their primary treating hematologist. After verbal consent was obtained, their contact numbers were sourced from the databases of the Maternity and Children Hospital and the Hereditary Blood Diseases Centre in Al-Ahsa. The primary caregivers were eligible to participate in this study if they had a child with SCD. Exclusion criteria included parents who were receiving mental health care services or if their child was above 18 years of age.

### 2.2 Procedures

Prior to participation, all participants were provided with detailed information about the study objectives, procedures, potential risks, and benefits. Informed consent was obtained from all participants before any data collection commenced. Participants were assured of their anonymity and confidentiality. A structured interview was conducted with 142 caregivers over the phone, while 151 caregivers participated through an online Google form questionnaire. The two methods were used to identify any significant difference between them, especially after the COVID-19 era, when reduced contact became more common in contemporary research practices. The response rate for the phone calls was 80 %. Each phone call took 22–27 min to answer all the questions. Demographic information included questions about the level of education of the primary caregiver, age, family income, and the type of therapy coverage.

An Arabic version of the Impact of Events Scale-Revised, developed by Daniel Weiss and Charles Marmar and translated and validated by Ali (2022), was used to diagnose PTSD according to DSM-IV [17]. The primary caregivers were asked to rate the presence of each of the 22 statements related to PTSD on a 4- scale ranging from "not at all" [0] to "extremely" [4]. Questions were formulated to be related to the sickle cell disease of their child to ensure assessment of SCD-related PTSD. For example, item 1 asked whether there was any reminder that brought back feelings about the disease or the treatment of the child. Total IES-R scores range from 0 to 88, with higher scores representing a high probability of PTSD presence.

An Arabic version of the Post-traumatic growth inventory developed by Lawrence Calhoun and Richard Tedeschi and translated and validated by Al-Nasah, was also used to assess the growth and self-improvement a person undergoes after trauma [18]. The participants were asked if they experienced 21 different items related to growth on a 6-point scale ranging from "I did not experience this as a result of my crisis" [0] to "I experienced this change to a very great degree as a result of my crisis" [5]. A high total score indicates that the person has undergone a positive change.

### 2.3 Data management and statistical analyses

The data was stored in Excel sheets and reviewed. Then, it was analyzed using the statistical package SPSS, release 26 (SPSS Inc., Chicago, IL). All analyses were performed with two-sided tests; p < 0.01, and p < .05 were considered significant. In demographic data such as gender distribution and family income, descriptive statistics such as frequencies, mean, median, standard deviation, minimum, and maximum values were used, as well as in IES-R dimensions and PTGI. ANOVA test was also used to analyze the differences between the mean scores of IES-R and PTGI scales of demographic data, such as the different family income groups. Tukey's HSD test was used to see the significance of the means of different groups of demographics in relation to IES-R score. Pearson correlation coefficient was used to examine the relationship between the three dimensions of IESR (intrusion, avoidance, and hyperarousal) and the five dimensions of post-traumatic growth in the PTGI (Relating to others, New possibilities, Personal strength, Spiritual change, and an Appreciation of life), as well as the relationship between PTSD, demographics, and illness-related variables.

## 3 Results

The overall sample was composed of 294 caregivers. The details of the sociodemographics are shown in Table 1a, Table 1ba, b 49.2 % of the responses were gathered online, while 50.8 % came from phone interviews. Mothers were the primary caregivers in 54.4 % of the sample, while fathers were 44.9 %, and others were barely 0.6 %. Regarding the education level, 24.7 % of the caregivers were below secondary school, 35.8 % had completed secondary school, and 39.6 % had a university degree. Most of the caregivers were in an age group between 41 years and 50 years (40.8 %). Most of the sample had a family income from 8000 to 12000 SR (32.3 %) and an income of less than 5000 SR (29.4 %).

**Table 1a:** Sociodemographic data of the caregivers of children with sickle cell disease (n = 315).

Characteristics	No.	%
Primary caregiver		
Mother	172	54.4
Father	142	44.9
Others	2	0.6
Educational level		
Below secondary school	78	24.7
Secondary school	113	35.8
University degree	125	39.6
Age of the caregiver		
20–30	28	8.9
31–40	118	37.3
41–50	129	40.8
51–60	41	13
Age of the partner		
20–30	19	6
31–40	108	34.2
41–50	140	44.3
51–60	38	12
Single parent	11	3.5
Family income (by Saudi Riyal)		
Less than 5K	93	29.4
5K–7K	52	16.5
8K–12K	102	32.3
13K–16K	47	14.9
More than 16K	22	7
Therapy payment		
Fully covered	294	93
Cash contribution	22	7
Chronic disease		
Sickle cell disease	44	14.0
One chronic disease	51	16.2
Multiple chronic disease	26	8.3
Multiple chronic disease including Sickle cell disease	27	8.6
None	168	53.3
Mental disorder		
Yes	22	7
No	294	93
Substance use		
Smoking	68	21.5
Morphine	2	0.6
None	246	77.8
Number of children		
1 child	44	13.9
Two	65	20.6
Three	61	19.3
Four	61	19.3
Five	28	8.9
More than 5	57	18
Do you attend your child appointments?		
Always	276	87.3
Sometimes	39	12.3
No	1	0.3
Do you think that you receive family support for your child's disease?		
Yes	153	48.4
A little	89	28.2
No	74	23.4
Do you have a relative with the same disease?		
Yes	261	82.6
No	55	17.4

**Table 1b:** Characteristics of the children with sickle cell disease (n = 315).

Characteristics	No.	%
Gender		
Male	227	71.8
Female	89	28.2
The age of the child when they're informed with the diagnosis.		
Birth to 2 years	188	59.5
3–6 years	104	32.9
6–9 years	17	5.4
10–13 years	7	2.2
Having a complication of SCD		
Multiple complications	18	5.7
One complication	68	21.5
No complications	230	72.8
History of surgeries		
Multiple surgeries	6	1.9
One surgery	77	24.4
No surgeries	211	66.98
Hospital admissions because of SCD?		
Once	120	38
Twice	29	9.2
Three times	49	15.5
Four times	24	7.6
Five times	9	2.8
More than 5 times	85	26.9
Does the child have another hereditary disease?		
G6PD deficiency	63	19.9
Thalassemia	24	7.6
None	229	72.5

In terms of the differences between the phone calls and the online questionnaires, the results in Table 2, Table 3 indicated significant differences in the mean of PTG scores between them, with online participants having higher mean score (M = 63.09 ± 27.99) than phone calls participants (M = 36.12 ± 25.24), p < 0.001. Similarly, trauma levels, as measured by IES-R, were significantly higher in the online group (M = 12.32 ± 13.24) compared to the phone calls group (M = 9.15 ± 10.46), p = 0.023.

**Table 2:** Independent samples t-test comparing the means of IESR scores between online and phone interview data collection modes.

Independent samples *t*-test for the means of IESR score						95 % Confidence Interval of the Difference		
Mode of Collection	N	Mean ± SD of IESR score	p-value. (2-tailed)	t-statistic	df	Mean Difference	Lower	Upper
Online	142	12.32 ± 13.24	0.023	2.29	291	3.18	0.44	5.91
Phone Interviews	151	9.15 ± 10.46

**Table 3:** Independent samples t-test comparing the means of PTGI scores between online and phone interview data collection modes.

Independent samples *t*-test for the means of PTGI score						95 % Confidence Interval of the Difference		
Mode of Collection	N	Mean ± SD of PTGI score	p-value. (2-tailed)	t-statistic	df	Mean Difference	Lower	Upper
Online	142	63.09 ± 27.99	0.000	8.67	291	26.98	20.85	33.09
Phone Interviews	151	36.12 ± 25.24						

Table 4 shows the impact of event score among the primary caregivers consisting of mothers, fathers, and, in some children, other individuals who took care of them. It includes information on the mean, standard deviation, 95 % confidence intervals for the mean, ANOVA results, and Tukey's HSD test results. The average score for mothers (n = 158) was 13.10 (SD = 13.09), with a 95 % confidence interval ranging from 11.11 to 15.23. For fathers (n = 134), the average score was 7.81 (SD = 9.8), with a 95 % confidence interval ranging from 6.13 to 9.48. The gender of the caregiver had a significant effect on the total score of the IESR scale F [2,291] = 7.63, p = 0.001. Moreover, the Tukey HSD post-hoc analysis results indicated a significant mean difference in IES scores between mothers and fathers (Mdiff = 5.36, SE = 1.37, p < 0.001).

**Table 4:** ANOVA results comparing the means of IESR scores among different primary caregivers (mother, father, others), and Tukey's HSD test results.

95 % Confidence Interval for Mean ANOVA							
Primary Caregiver	N	Mean ± SD of IESR score	Lower Bound	Upper Bound	F-value	p-value	Tukey's HSD Test Results
Mother	158	13.10 ± 13.09	11.11	15.23	7.633	0.001	vs. Father: p = 0.000
Father	134	7.81 ± 9.8	6.13	9.48			vs. Others: p = 0.962
Others	2	10.00 ± 11.31	−91.65	111.65			vs. Mothers: p = 0.923
Total	294	10.70 ± 11.96	9.33	12.08			

*The mean difference is significant at the 0.05 level.

Regarding the income level, the average score of the IES was the highest for the lowest income range (less than 5000 SR), with a mean of 16.17. The average score decreases as income increases, with the lowest mean score of 5.73 for the income range of 13000–16000 SR. Moreover, Table 5 shows that the results of an ANOVA test revealed a significant difference in the IES score between the means of the income categories F(4, 289) = 9.442, p < 0.001. Pairwise comparisons of the means using Tukey HSD revealed significant differences between families with less than 5000SR income and almost all of the other categories. More specifically, those who had a family income of less than 5000 SR (M = 16.17, SD = 14.65), had significantly higher average IES scores than those who had a family income of 8000 SR to 12000 SR (M = 8.30, SD = 8.18, p < .001), 13000 to 16000 (M = 5.73, SD = 8.50, p p < .001), and those who had an income that was more than 16000 SR (M = 5.81, SD = 7.51, p = 0.002).

**Table 5:** ANOVA results comparing the means of IESR scores among different family income groups, and Tukey's HSD test results.

95 % Confidence Interval for Mean ANOVA							
Family income	N	Mean ± SD of IESR score	Lower Bound	Upper Bound	F-value	p-value	Tukey's HSD Test Results
less than 5000 SR	84	16.17 ± 14.65	12.99	19.35	9.44	. 000	Vs 5000 to 7000 SR: p = 0.391 Vs 8000 to 12000 SR: p = 0.000 Vs 13000 to 16000 SR: p = 0.000 Vs More than 16000 SR: p = 0.002
5000 to 7000	50	12.58 ± 13.33	8.79	16.37			Vs 8000 to 12000 SR: p = 0.198 Vs 13000 to 16000 SR: p = 0.029 Vs More than 16000 SR: p = 0.148
8000 to 12000 SR	94	8.30 ± 8.18	6.62	9.97			Vs 13000 to 16000 SR: p = 0.722 Vs More than 16000 SR: p = 0.893
13000 to 16000	45	5.73 ± 8.50	3.18	8.29			Vs More than 16000 SR: p = 1.000
More than 16000	21	5.81 ± 7.51	2.39	9.23			Vs 13000 to 16000 SR: p = 1.000
Total	294	10.70 ± 11.96	9.33	12.08			

*The mean difference is significant at the 0.05 level.

In terms of the education level, the detailed descriptive statistics are presented in Table 6. The mean scores for IESR were highest for those with below secondary school education (M = 15.89), followed by secondary school education (M = 11.09), and lowest for those with a university degree (M = 7.60). The ANOVA test showed a statistically significant difference in the mean scores of IESR based on level of education F (2,291) = 10.993, p < .001. The Tukey HSD test further showed that the mean score for those with a university degree was significantly lower than those with below secondary school education (p < .001) and secondary school education (p = 0.024).

**Table 6:** ANOVA results comparing the means of IESR scores among different levels of education of the primary caregiver, and Tukey's HSD test results.

95 % Confidence Interval for Mean ANOVA							
Level of Education of the primary care giver	N	Mean ± SD	Lower Bound	Upper Bound	F-value	p-value	Tukey's HSD Test Results
Below secondary school	65	15.89 ± 14.71	12.25	19.54	10.993	0.000	Vs Secondary School p = 0.024
Secondary School	107	11.09 ± 11.39	8.91	13.28			Vs University Degree: p = 0.060
University Degree	122	7.60 ± 9.68	5.86	9.33			Vs Below secondary school: p = 0.000
Total	294	10.70 ± 11.96	9.33	12.07			

*The mean difference is significant at the 0.05 level.

Regarding the effect of the child having a complication of SCD on the parent's IESR scores, the results in Table 7 shows that the mean IESR score of parents who their child had one complication (15.88) was similar to those who had a child with multiple complications (15.00). On the other hand, parents with a child who did not experience any complications had the lowest mean score (8.91). As a result, it was clear that the effect of complications on the parents' IESR score is significant F(2,291) = 9.782, p < .001.

**Table 7:** ANOVA results comparing the means of IESR scores based on having complications of SCD (one complication, no complications, multiple complications), and Tukey's HSD test results.

95 % Confidence Interval for Mean ANOVA							
Having a complication of SCD	N	Mean ± SD of IESR score	Lower Bound	Upper Bound	F-value	p-value	Tukey's HSD Test Results
Had one complication	60	15.88 ± 13.19	12.48	19.29	9.78	. 000	Vs No complications: p = 0.000
No complications	216	8.90 ± 11.25	7.40	10.42			Vs Multiple complications: p = 0.084
Multiple complications	18	15.00 ± 10.27	9.89	20.11			Vs Had one complication p = . 957
Total	294	10.70 ± 11.96	9.33	12.08			

*The mean difference is significant at the 0.05 level.

Regarding the history of surgeries in Table 8, the mean IESR score for parents of children who had multiple surgeries (11.00) was higher than that of those who did not have surgeries (9.36) but lower than that of those with at least one surgery (14.35). Moreover, the Anova results showed that surgeries significantly affect the mean IESR score (F = 5.041, p = .007).

**Table 8:** ANOVA results comparing the means of IESR scores based on the history of surgeries (at least one surgery, no surgeries, multiple surgeries), and Tukey's HSD test results.

95 % Confidence Interval for Mean ANOVA							
Doing surgeries	N	Mean ± SD of IESR score	Lower Bound	Upper Bound	F-value	p-value	Tukey's HSD Test Results
Had at least one surgery	77	14.35 ± 12.53	11.51	17.20	5.04	0.007	Vs Didn't have a surgery: p = 0.005
Didn't have a surgery	211	9.36 ± 11.58	7.79	10.94			Vs Had multiple surgeries: p = 0.940
Had multiple surgeries	6	11.00 ± 8.99	1.57	20.40			Vs Had at least one surgery p = 0.781
Total	294	10.70 ± 11.96	9.33	12.08			

*The mean difference is significant at the 0.05 level.

In terms of having another hereditary disease, the results in Table 9 revealed that parents whose child had thalassemia had the lowest mean IESR score (8.76), followed by parents whose child did not have another hereditary disease (10.07), and parents whose child had G6PD (13.78). However, the ANOVA table indicated that the differences in the means between the groups were not statistically significant F(2,291) = 2.524, p = 0.082.

**Table 9:** ANOVA results comparing the means of IESR scores based on the child having other hereditary diseases (G6PD, none, Thalassemia), and Tukey's HSD test results.

95 % Confidence Interval for Mean ANOVA							
The child having other hereditary diseases	N	Mean ± SD of IESR score	Lower Bound	Upper Bound	F-value	p-value	Tukey's HSD Test Results
G6PD	58	13.78 ± 13.42	10.25	17.30	2.52	0.082	Vs None: p = 0.090
None	215	10.07 ± 11.50	8.52	11.61			Vs Thalassemia: p = 0.881
Thalassemia	21	8.76 ± 11.41	3.57	13.95			Vs G6PD: p = 0.224
Total	294	10.70 ± 11.96	9.33	12.08			

Regarding the effect of admission rate, the results in Table 10 show that the mean IESR score for all participants was (M = 10.7041 ± 11.95687). Participants who had their children admitted to the hospital more than five times had the highest mean IESR score (17.0845), while those who had their children admitted only once had the lowest mean score (5.5169). Furthermore, the results of the ANOVA test indicated a statistically significant difference between the means of IESR scores for different admission rates F(5,288) = 10.688, p < .001. In Tukey's HSD test, the differences were statistically significant for all comparisons except for the comparison between twice and three times hospital admissions and between five times and more than five hospital admissions.

**Table 10:** ANOVA results comparing the means of IESR scores based on the frequency of hospital admissions due to SCD, and Tukey's HSD test results.

95 % Confidence Interval for Mean ANOVA							
Frequency of Hospital admissions because of SCD	N	Mean ± SD	Lower Bound	Upper Bound	F-value	p-value	Tukey's HSD Test Results
Once	118	5.52 ± 7.71	4.11	6.92	10.688	. 000	Vs Twice: p = 0.390 Vs Three times: p = 0.005 Vs Four times: p = 0.013 Vs Five times: p = 0.276 Vs More than five times: p = 0.000
Twice	29	9.93 ± 13.09	4.95	14.91			Vs Three times: p = 0.927 Vs Four times: p = 0.792 Vs Five times: p = . 933 Vs More than five times: p = 0.042
Three times	45	12.49 ± 11.56	9.02	15.96			Vs Four times: p = 0.996 Vs Five times: p = 0.999 Vs More than five times: p = 0.252
Four times	23	13.91 ± 8.62	10.20	17.64			Vs Five times: p = 1.000 Vs More than five times: p = 0.840
Five times	8	14.13 ± 10.06	5.71	22.54			Vs More than five: p = 0.980
More than five times	71	17.08 ± 14.81	13.58	20.59			Vs Five times: p = 0.980
Total	294	10.70 ± 11.96	9.33	12.08			

*The mean difference is significant at the 0.05 level.

Table 11 shows the descriptive statistics of the IES-R and PTGI scores and their subscales. The mean score for IES-R was 10.70, indicating that the caregivers experienced some level of stress related to their role as caregivers. The IES-R subscales included intrusive thoughts, avoidance, and hyperarousal. The mean scores for these subscales were 3.96, 3.55, and 3.19, respectively. On the other hand, the mean score for PTGI was 49.21, indicating that caregivers also experienced some level of post-traumatic growth. PTGI is composed of five subscales. They relate to others, new possibilities, personal strength, spiritual change, and appreciation of life. The mean scores for these subscales were 14.22, 10.18, 11.67, 5.86, and 7.28, respectively.

**Table 11:** Mean and standard deviation (SD) of IESR and PTGI scores, including the subscales.

Characteristics	Mean ± SD
IES	
PTSD_intrusive thoughts	3.96 ± 4.17
PTSD_Avoidance	3.55 ± 4.56
PTSD_Hyperarousal	3.19 ± 3.86
*Total IES score*	10.7 ± 11.96
PTGIT score	
PTGI_ Relating to others	14.22 ± 10.05
PTGI_ New Possibilities	10.18 ± 8.31
PTGI_ Personal Strength	11.67 ± 6.54
PTGI_ Spiritual Change	5.86 ± 3.47
PTGI_ Appreciation of Life	7.28 ± 4.46
*PTGIT total score*	49.21 ± 29.75

In terms of the relationships between the three elements of PTSD (intrusion, avoidance, and hyperarousal), Table 12a shows that there were positively strong relationships between them. For example, the Pearson correlation coefficient of intrusive thoughts and avoidance was found to be strongly positive and statistically significant (r = . 849, p = 0.000). Similarly, the correlation of avoidance and hyperarousal was found to be as strongly positive and significant (r = . 849, p = 0.000). Moreover, the correlation of hyperarousal and intrusive thoughts was found to be stronger than the formers (r = . 855, p = 0.000).

**Table 12a:** Pearson Correlation between the total scores of IESR and PTGI.

		IES_R	PTGI
IES_R	Pearson Correlation	1	0.328^a^
	Sig. (2-tailed)		0.000
	N	294	294
PTGIT	Pearson Correlation	0.328^a^	1
	Sig. (2-tailed)	0.000	
	N	294	294

a Correlation is significant at the 0.01 level (2-tailed).

In addition, the five dimensions of post-traumatic growth in the PTGI (Relating to others, New Possibilities, Personal Strength, Spiritual Change, and Appreciation of Life) were positively and strongly correlated (Table 12b). The strongest degree of positive correlation was seen between relating to others and openness to new possibilities (r = . 843, p = 0.000), followed by the positively strong correlation between openness to new possibilities and personal strength (r = . 807, p = 0.000). On the other hand, the weakest degree of positive correlation among them was detected between the spiritual change and appreciation of life (r = . 682, p = 0.000).

**Table 12b:** Pearson Correlation between the subdivisions of the IESR scale.

		PTSD_INTRUSIVE THOUGHT	PTSD_ Avoidance	PTSD_ Hyperarousal
PTSD_ Intrusive thought	Pearson Correlation	1	0.849^a^	0.855^a^
	Sig. (2-tailed)		0.000	0.000
	N	294	294	294
PTSD_ Avoidance	Pearson Correlation	0.849^a^	1	0.849^a^
	Sig. (2-tailed)	0.000		0.000
	N	294	294	294
PTSD_ Hyperarousal	Pearson Correlation	0.855^a^	0.849^a^	1
	Sig. (2-tailed)	0.000	0.000	
	N	294	294	294

a orrelation is significant at the 0.01 level (2-tailed).

Finally, regarding the relation between the degree of the trauma score in the Impact of Event Scale (IES-R) and the post-traumatic growth in the Post-Traumatic Growth Inventory (PTGI), a Pearson correlation coefficient in Table 12c indicated a moderately positive correlation between them (r = . 328, p = 0.000).

**Table 12c:** Pearson Correlation between the subdivisions of the PTGI scale.

		PTGI_RTO	PTGI_NP	PTGI_PS	PTGI_SC	PTGI_AOL
PTGI_ Relating to others	Pearson Correlation	1	0.843^a^	0.751^a^	0.704^a^	0.684^a^
	Sig. (2-tailed)		0.000	0.000	0.000	0.000
	N	294	294	294	294	294
PTGI_ New Possibilities	Pearson Correlation	0.843^a^	1	0.807^a^	0.727^a^	0.770^a^
	Sig. (2-tailed)	0.000		0.000	0.000	0.000
	N	294	294	294	294	294
PTGI_ Personal Strength	Pearson Correlation	0.751^a^	0.807^a^	1	0.787^a^	0.798^a^
	Sig. (2-tailed)	0.000	0.000		0.000	0.000
	N	294	294	294	294	294
PTGI_ Spiritual Change	Pearson Correlation	0.704^a^	0.727^a^	0.787^a^	1	0.682^a^
	Sig. (2-tailed)	0.000	0.000	0.000		0.000
	N	294	294	294	294	294
PTGI_ Appreciation of Life	Pearson Correlation	0.684^a^	0.770^a^	0.798^a^	0.682^a^	1
	Sig. (2-tailed)	0.000	0.000	0.000	0.000	
	N	294	294	294	294	294

a Correlation is significant at the 0.01 level (2-tailed).

## 4 Discussion

This study aimed to measure the level of trauma and its effects on primary caregivers of children with Sickle cell disease in AlAhsa, Saudi Arabia, using the IESR and PTGI scales and their relationship to each other.

It included 294 participants, with mothers accounting for 54.4 % of the sample and fathers accounting for 44.9 % (Table 1a). Regarding recruitment, most responses (50.8 %) were gathered through phone interviews, while (49.2 %) were collected through a direct online invitation after a verifying call. Most caregivers had a university degree, were between the ages of 41 and 50, and had a family income ranging from 8000 to 12000 SR (Table 1a). Regarding the health aspect of the caregivers, most were not suffering from chronic diseases and were not using mental health services (Table 1a).

Using the IERS and PTGI scales, our study revealed that the caregivers experienced some level of stress related to the event, i.e., their child's diagnosis and situation. Also, many caregivers experienced some level of post-traumatic growth as a result of their trauma, such as a shift in their priorities. Furthermore, the trauma may have had a profound impact on their spiritual beliefs and practices. In terms of PTSD elements (intrusive thoughts, avoidance, and hyperarousal), the mean scores were 3.96, 3.55, and 3.19, respectively, with positive correlations between all of them (Table 11, Table 12bb). Furthermore, it is worth mentioning that the highest positive correlation was between hyperarousal and intrusive thoughts (r = . 855, p = 0.000) (Table 12b).

Regarding the relationship between IERS and PTGI, our research showed a moderately positive correlation (r = . 328, p = 0.000) (Table 12a). There are several studies discussing the effects of chronic diseases by their different phases (diagnosis, treatment, and late complications) on the traumatic experience of parents as caregivers for their children. For example, three studies have been conducted on parents of cancer survivor children showed that diagnosis and treatment of cancer can have traumatic effects on caregivers, which manifest in PTSD symptoms in the long run [[19], [20], [21]].

Furthermore, caregivers addressed intrusive thoughts and distress as reminders of the diagnosis and treatment of cancer [19]. These findings are consistent with our study regarding SCD. In terms of studies on SCD in children and caregivers, intrusive thoughts, which has the highest mean score in our study, have been shown to worsen the consequences of vasoccclusion, increasing children's feeling of pain and causing doctors to prescribe analgesics inadequately instead of providing psychological support [22], (Table 11).

Our study has found many factors affecting the IERS score. First, the gender of the caregiver had a significant effect on the total score of the IESR scale, with a higher average score (13.17) among mothers (Table 4). Such a finding is consistent with what has been found in the literature that mothers are more prone to Post-traumatic Stress Syndrome (PTSS) early in the disease and PTSD later [1]. In their study of caregivers of children with epilepsy, Carmassi et al. discovered that 13.3 % of mothers developed PTSD while only 4.5 % of fathers did. When the diagnosis threshold was lowered, 43.3 % of mothers had partial PTSD compared to 25 % of fathers [23]. These differences between mothers and fathers can be explained by the fact that males and females have different coping mechanisms; however, in other studies, lower household income and higher disease-related costs were found to be associated with a higher prevalence of PTSD among fathers [24].

Second, the IESR's average score was highest for the lowest income range (less than 5000 SR) (Table 5). This finding is consistent with many studies' findings that low income and high illness-related expenses are important contributors to caregivers' degree of distress [25]. Moreover, it was found that fathers were primarily affected by these factors [24].

Third, in terms of the effect of education level on IERS score, it is safe to assume that education is a protective factor since the mean scores for IESR were highest for those with less than secondary school education (Table 6). Moreover, a study conducted in Egypt on 96 caregivers found that higher professional status and education levels were negatively associated with PTSS prevalence [26].

Fourth, we discovered that parents of children with Sickle cell disease, who had higher rates of admission, had higher trauma scores (Table 10). There is no data on the rate of admissions and the prevalence of PTSD; however, many studies correlated the hospital's length of stay and PTSD. For example, in burn victim children and transplant candidates, the length of hospital stay was correlated with PTSS and PTSD in their caregivers [27]. The length of stay effect could be a confounder for many other findings in our data, such as the higher IESR scores among parents of children who underwent multiple surgeries or had multiple complications.

Finally, our findings suggest that the method of data collection can influence participants' levels of post-traumatic growth (PTG) and the trauma degree. When compared to phone calls, online participants reported significantly higher levels of PTG (P < 0.001) and trauma (P = 0.023) (Table 2). In light of the testimonies of the data collectors, these differences must be attributed to a variety of factors extending beyond the mere convenience and privacy associated with online questionnaires. They reported frequent instances of grief and tears during phone calls, along with repeated requests for clarifications regarding terms like "numb feelings.". Therefore, researchers in trauma-related research should exercise caution and seek a source to verify the accuracy of the collected data.

Despite the fact that we put every effort into coming up with reliable and generalizable data, our study has a few limitations. Firstly, although we tried to enrol participants from different canters, most of the caregivers were following the hematology clinic at the MCH hospital in Alahsa City. Secondly, and due to its nature, this is a cross-sectional study that is prone to a recall bias and a limited ability to deduce a temporal relationship between PTSD elements, PTGI elements, and sociodemographic factors. So, a follow-up study is recommended to confirm the temporal relationship. Finally, 294 participants were enough for most of the measured factors. However, the raw data showed promising important factors that were not statistically significant due to the lack of sufficient participation and institutional support for the research team.

However, to the best of our knowledge, no local similar studies have explored the degree of distress among caregivers of children with SCD. So, our study aimed to measure the effect of Sickle cell disease in children in AlAhsa, Saudi Arabia, on their caregivers in order to identify the most vulnerable group and provide a well-designed intervention to improve their quality of life.

## 5 Conclusion

In conclusion, similar to parents of children with cancer, caregivers of children with SCD experienced intrusive thoughts and distress. Several sociodemographic factors influenced the level of trauma experienced by caregivers. Mothers exhibited higher levels of trauma compared to fathers, possibly due to differences in coping mechanisms. Education level appeared to be a protective factor, with higher education levels associated with lower trauma scores. The study also revealed a correlation between the child's rate of hospital admissions and caregiver trauma scores. Longer hospital stays were associated with increased distress among caregivers, suggesting the need for support during hospitalization. While the study provides valuable insights, it has limitations, including a limited sample from a specific clinic and a cross-sectional design. Future research should consider longitudinal studies to establish temporal relationships.

## CRediT authorship contribution statement

**Ali Alsaad:** Writing – review & editing, Supervision, Investigation, Conceptualization. **Abdullah Alghanim:** Writing – review & editing, Writing – original draft, Validation, Supervision, Software, Resources, Project administration, Methodology, Investigation, Formal analysis, Conceptualization. **Mohammed Aldawood:** Writing – review & editing, Writing – original draft, Validation, Supervision, Project administration, Conceptualization. **Ali Al Zaid:** Writing – original draft, Investigation, Data curation. **Hussain Aldehneen:** Writing – original draft, Project administration, Investigation, Data curation. **Rawan Aldrees:** Writing – original draft, Data curation, Conceptualization. **Ammar Alsalem:** Writing – review & editing, Validation, Methodology, Investigation. **Sami Albattat:** Writing – review & editing, Resources, Funding acquisition, Data curation, Conceptualization. **Abbas Al Mutair:** Writing – review & editing, Supervision, Project administration, Methodology, Investigation, Formal analysis, Conceptualization.

## Declaration of competing interest

The authors declare that they have no known competing financial interests or personal relationships that could have appeared to influence the work reported in this paper.
